# Editorial: Cellular and molecular determinants of pregnancy success at the fetal-maternal interface in health and disease

**DOI:** 10.3389/fcell.2023.1240481

**Published:** 2023-07-14

**Authors:** Peeyush K. Lala, Charles H. Graham

**Affiliations:** ^1^ Departments of Anatomy and Cell Biology and Oncology and Children’s Health Research Institute, Schulich School of Medicine and Dentistry, London, ON, Canada; ^2^ Department of Biomedical and Molecular Sciences, School of Medicine, Faculty of Health Sciences, Queen’s University, Kingston, ON, Canada

**Keywords:** feto-maternal interface, preeclampsia, fetal growth restriction, metabolic adaptations in pregnancy, microRNAs in pregnacy, exosomes in pregnancy, decorin, osteopontin

**FIGURE 1:**
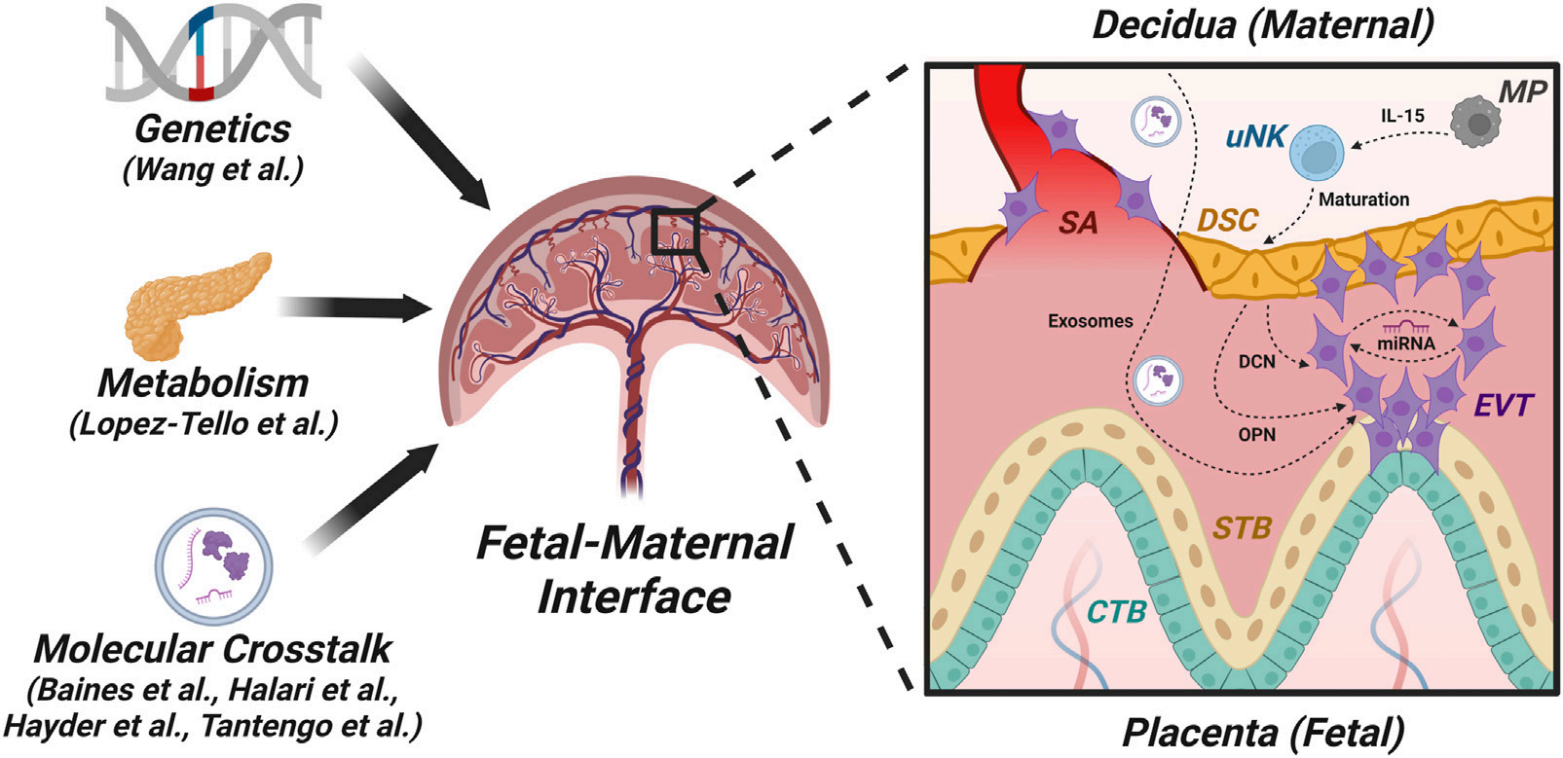
Factors and pathways in the fetal-maternal interface. This figure is a graphic representation of the structure of the first-trimester human placenta after the formation of chorionic villi showing the fetal-maternal interface and some of the players engaged in fetal-maternal cross-talk. The fetal chorionic villi include different trophoblast sub-populations: cytotrophoblasts (CTBs) containing trophoblast stem cells, which give rise to syncytiotrophoblast (STB) by cell fusion and extravillous trophoblasts (EVTs) that invade the maternal decidua, which consists of decidualized stromal cells (DSCs). EVTs also invade the spiral arteries (SAs) and participate in the transformation of these arteries into low-resistance, high-flow tubes to carry adequate maternal arterial blood for fetal nourishment. Impairment of this blood flow leads to placental dysfunction that can result in fetal growth restriction (FGR) and preeclampsia (PE) in the mother. One major mechanism in fetal-maternal crosstalk is via exosomes, which can contain microRNAs (miRNAs). For example, DSCs produce decorin (DCN), which can compromise EVT functions independently or through certain miRNAs. Additionally, macrophages (MPs) produce IL-15, which acts on uterine natural killer (uNK) cells that promote the maturation of DSCs. These DSCs secrete osteopontin (OPN), which modulates EVT functions.

Pregnancy in placental mammals, including humans, starts with the process of implantation of the blastocyst, followed by placental development. Prior to implantation, primitive trophectoderm serves as the earliest source of trophoblast stem cells (TSC). After implantation, the developing placenta serves multiple functions essential for fetal growth and survival. The hemochorial type of placenta, such as in the human, is a highly invasive tumor-like structure that invades the pregnant uterus and its vasculature to tap on the maternal blood and nourish the fetus. Placental functions are provided by two classes (villous and extravillous) of trophoblast cells. They originate from the bi-potent TSC contained within the cytotrophoblast (CTB) layer of the chorionic villi. The villous syncytiotrophoblast (STB) layer lining the maternal blood sinusoids arise from the TSC by cell fusion and is primarily engaged in absorptive, exchange and endocrine functions. Microvilli on the STB surface enormously increase the surface area of the STB to amplify functional competence. Extravillous trophoblast (EVT) cells arise from the TSC as highly migratory cell columns, which proliferate at the villus base and invade the uterine decidua and spiral arteries (SA). They undergo endothelial-like (endovascular) differentiation to invade and remodel distal segments of the SA into low-resistance tubes that allow a steady flow of maternal blood for fetal nourishment (Figure 1). Poor EVT invasion and SA remodeling are linked with maternal preeclampsia (PE) and fetal growth restriction (FGR). A large number of molecules produced locally at the fetal-maternal interface, either by the trophoblast or various maternal cell types within the endometrium or the decidua (decidual cells, immune cells, stromal cells), positively or negatively regulate trophoblast differentiation, proliferation, migration and invasion, and other pregnancy-related events such as decidual maturation, angiogenesis and lymphangiogenesis, maintaining healthy utero-placental homeostasis. Molecules responsible for utero-placental homeostasis include a variety of growth factors (e.g., Hb-EGF, IGF-1, IGF-II, VEGF, PLGF, HGF), growth factor-binding proteins (e.g., IGF-BPs) and proteoglycans (e.g., decorin, biglycan), sialoprotein (e.g., osteopontin), cytokines (e.g., IL6, IL-15), chemokines (e.g., CX3CL1, CCL14, and CCL4), lipid derivatives (e.g., PGE2) and matrix-degrading enzymes (e.g., MMPs, serine and cysteine proteases, uPA) or their inhibitors (e.g., TIMPs, PAI-1, PAI-2) (Reviewed by Lala and Nandi, 2016). Altered production of some of these factors can lead to a variety of pregnancy-related pathologies such as pregnancy loss, FGR, PE and hyper-invasive placentas (placenta accreta, increta and percreta). A good deal of information is already available on the functions of specific maternal or fetally-derived cells of the placenta and their products responsible for normal placental functions and their alterations in the pathogenesis of pregnancy-associated diseases. However, there remains a large gap in this knowledge. The present Research Topic aims to review specific areas of cellular/molecular interactions that contribute to normal pregnancy or pregnancy-associated diseases and identify gaps in the knowledge for future research to address.

A call for submission of original articles and reviews resulted in eight submissions, of which six were accepted having gone through the peer review process. They are summarized below. They include roles of genetic variations on fetal-placental development, metabolic adaptations during pregnancy, roles of extracllular vesicles (exosomes) and their cargos such as microRNAs in fetal-maternal communications in health and disease, and the roles of IL-15 in the production of osteopontin, a decidual product which regulates trophoblast invasion.

Wang et al. (University of British Columbia, Vancouver, Canada) reviewed and provided original data on genetic variations responsible for placental insufficiency in pregnancy. Genetic variation shapes placental development and function, which has long been recognized to impact fetal growth and adverse pregnancy outcomes such as miscarriage or maternal preeclampsia. Early epidemiological studies provided evidence for a strong heritable component to these conditions with contributions by both maternal and fetal-placental genes. Subsequently, cytogenetic studies of the placenta and the advent of prenatal diagnosis to detect chromosomal abnormalities provided direct evidence for the importance of spontaneously arising genetic variation in the placenta, such as trisomy and uniparental disomy, drawing inferences that remain relevant to this day. Candidate gene approaches highlighted the roles of genes influencing immune interactions at the maternal-fetal interface and angiogenic factors. More recently, the emergence of molecular techniques, particularly high-throughput technologies, such as Single-Nucleotide Polymorphism (SNP) arrays, has facilitated the discovery of copy number variations and the study of SNP associations with conditions related to placental insufficiency. This review integrates past and more recent knowledge to provide important insights into the role of placental function on fetal and perinatal health, as well as into the mechanisms leading to genetic variations during development.

Pregnancy requires adaptations in maternal metabolism that promote glucose and fatty acid availability to support fetal growth. Lopez-Tello et al. (University of Cambridge, Cambridge, United Kingdom) provided original data in support of the notion that the PI3K-p110alpha signalling pathway is needed for maternal metabolic adaptations to pregnancy. The phosphoinositol-3-kinase (PI3K) signalling pathway controls multiple biological processes, and defects in this pathway are linked to multiple metabolic disorders, including insulin resistance and glucose intolerance in non-pregnant animals. However, relatively little is known about the contribution of PI3K signalling to maternal metabolic adaptations during pregnancy. Using mice with partial inactivation of the PI3K isoform, p110α (resulting from a heterozygous dominant negative mutation; Pik3ca-D933A), the effects of impaired PI3K-p110α signalling could be noted. Results reveal that non-pregnant mice lacking PI3K-p110α are glucose intolerant but exhibit compensatory increases in pancreatic glucose-stimulated insulin release and adipose tissue mitochondrial respiratory capacity and fatty acid oxidation. However, in pregnancy, mutant mice failed to show the normal increment in glucose intolerance and pancreatic β-cell mass observed in wild-type pregnant dams and exhibited further enhanced adipose tissue mitochondrial respiratory capacity. These maladaptations in pregnant mutant mice were associated with fetal growth restriction (FGR). Hence, the authors conclude that PI3K-p110α is a key regulator of metabolic adaptations that support fetal growth during normal pregnancy.

Exosomes are extracellular vesicles first described as such 30 years ago and have been implicated in cell–cell communication and the transmission of disease states and explored as a means of drug discovery. Yet fundamental questions about their biology remain unanswered. They serve as the cargo for a variety of molecules, including DNA, RNA, microRNA and proteins. Tantengco et al. (University of Texas, Galveston, United States) present original data to show that exosomes from *U. parvum (Ureaplasma parvum)*-infected ectocervical epithelial (ECTO) cells promote inflammation at the feto-maternal interface but are insufficient to cause preterm delivery. This study determined if exosomes from ECTO cells infected with *U. parvum* can carry bacterial antigens and cause inflammation at the feto-maternal interface using two organ-on-chip devices, one representing the vagina-cervix-decidua and another one mimicking the feto-maternal interface, and whether such inflammation can lead to preterm birth (PTB). Exosomes from *U. parvum*-infected ECTO cells were characterized using cryo-electron microscopy, nanoparticle tracking analysis, Western blot, and Exoview analysis. The antigenicity of the exosomes from *U. parvum*-infected ECTO cells was also tested using THP-1 cells and the authors’ newly developed vagina-cervix-decidua organ-on-a-chip (VCD-OOC) having six microchannel-interconnected cell culture chambers containing cells from the vagina, ectocervical, endocervical, transformation zone epithelia, cervical stroma, and decidua. The VCD-OOC was linked to the maternal side of their previously developed feto-maternal interface organ-on-a-chip (FMi-OOC). Cell culture media were collected after 48 h to determine the cytokine levels from each cell line via ELISA. For physiological validation of their *in vitro* data, high-dose exosomes from *U. parvum*-infected ECTO cells were delivered to the vagina of pregnant CD-1 mice on E15. Mice were monitored for preterm birth (PTB), showing that PTB was not induced by such delivery.

MicroRNAs (miRNAs) are an important cargo carried by exosomes. They constitute a large family of small non-coding RNAs (17-25 nucleotides) that typically downregulate target genes by repressing translation or facilitating the degradation of mRNAs. They are encoded by the genomes of most organisms. The human placenta expresses a distinct miRNA repertoire derived from the two largest clusters of miRNAs, the chromosome 14 miRNA cluster (C14MC) and the chromosome 19 miRNA cluster (C19MC). Although the functions of placental miRNAs are largely undefined, recent research has begun to shed light on their role in placental biology. Likewise, the finding that placental miRNAs are released into the maternal circulation via exosomes has raised the exciting prospect of using miRNA expression profiles as non-invasive markers of placental dysfunction. In this review, Hayder et al. (York University, Toronto, Canada) describes the events in miRNA biogenesis and summarize recent developments in our understanding of the biological action of miRNAs in the human placenta, with special reference to trophoblast invasion and spiral arterial remodelling and pathogenesis of preeclampsia.

Halari et al. (University of Western Ontario, London, Canada) provide original data showing that a decorin-induced, preeclampsia-associated microRNA-512-3p restrains extravillous trophoblast functions by targeting USF2/PPP3R1 axis. Decorin (DCN) is a leucine-rich proteoglycan produced by chorionic villus mesenchymal and decidual cells during human pregnancy. Studies from P.K. Lala’s laboratory demonstrated that decidua-derived DCN controls multiple trophoblast functions, including renewal, proliferation, migration, invasion and endovascular differentiation, mediated by DCN-binding to multiple tyrosine kinase receptors expressed by the trophoblast. Furthermore, DCN was shown to be selectively over-produced by the decidua in preeclampsia (PE) subjects and elevated in the second-trimester maternal plasma in PE before the appearance of clinical signs, presenting as a predictive biomarker for PE. The human placenta expresses many miRNAs, some of which are exclusively expressed by the trophoblast. Many of these miRNAs are dysregulated in PE-associated placentas, and some appear in the maternal blood as PE biomarkers. However, little is known about their contribution to the pathogenesis of PE, a multi-factorial disease associated with a hypo-invasive placenta. The objective of the present study was to examine whether exposure of extra-villous trophoblast (EVT) to DCN affects the expression of specific miRNAs and to test the role of these miRNAs in altering EVT functions. They identified miR-512-3p as one of the DCN-induced miRNAs that are also upregulated in PE placentas. It was shown to be elevated in ectopic DCN-over-expressing or exogenous DCN-treated first-trimester human trophoblast cell line HTR-8/SVneo. The use of miRNA-mimics and inhibitors revealed that miR-512-3p compromised trophoblast migration, invasion and VEGF-dependent endovascular differentiation. Finally, Protein Phosphatase 3 Regulatory Subunit B, Alpha (PPP3R1), a known target of miR-512-3p, was paradoxically elevated in miR-512-3p-overexpressing trophoblast and PE-associated placentas. Using Enrichr, a tool that consists of both a validated user-submitted gene list and a search engine for transcription factors, the authors found that PPP3R1 elevation resulted from the miR-512-3p binding to and targeting Upstream Transcription Factor 2 (USF2), which targeted PPP3R1. These findings reveal a novel aspect of the pathogenesis of PE and the biomarker potentials of this miRNA in PE.

Interleukin-15 (IL-15) is a ligand that activates human NK cells through components of the IL-2R in a pattern that is similar but not identical to that of IL-2. Unlike IL-2, IL-15 is produced by activated monocytes/macrophages. Osteopontin (OPN) is a highly phosphorylated sialoprotein with a high capacity to bind to calcium. It was first identified in 1985 by Franzén and Heinegård (1985) through isolation from the bovine bone matrix. OPN has an integrin-binding glycine-arginine-glycine-aspartic acid (GRGD) sequence in the central portion of the protein, which is highly conserved in all vertebrates examined. Baines et al. (University of Western Ontario, London, Canada) provide original data showing that the production of OPN, an important regulator of trophoblast invasion, is dependent on Interleukin-15 (IL-15) activity. Uterine Natural Killer (NK) cells are the predominant immune cells within the decidua during early pregnancy. These cells are thought to regulate aspects of decidualization and placental development, but their functions remain poorly characterized, especially in species with deeply invading trophoblasts, such as humans and rats. IL-15 is a cytokine required for NK cell development and survival. IL-15 mutant (IL15Δ/Δ) rats lack NK cells and exhibit altered placental development with precocious trophoblast invasion. In this study, they profiled gene expression differences between wild-type and IL15Δ/Δ implantation sites to reveal candidate factors produced by uterine NK cells that may regulate placentation and trophoblast invasion. There were 257 genes differentially expressed between wild-type and IL15Δ/Δ implantation sites on gestational day 9.5, including decreased expression of various NK cell markers in IL15Δ/Δ rats, as well as Spp1, which encodes OPN. In wild-type rats, OPN was present within the decidua basalis and adjacent to the primitive placenta, and OPN colocalized with the NK cell marker perforin. OPN was also detectable in uterine glands. Conversely, in IL15Δ/Δ rats, OPN and perforin were not readily detectable in the decidua despite robust OPN levels in uterine glands. Neutralization of OPN in media conditioned by cells isolated from the decidua decreased invasion of rat trophoblasts, suggesting that reduced levels of OPN alone is unlikely to account for the precocious trophoblast invasion in IL15Δ/Δ rats. Nonetheless, this study suggests that osteopontin is expressed by NK cells at the maternal-fetal interface in rats and may contribute to the modulation of trophoblast invasion.

It is hoped that this thematic publication will stimulate further research by placental biologists in adopting newer approaches for unravelling feto-maternal communications in health and disease. One example is building 3D organoids *in vitro* to include fetal and maternal cells that can be genetically manipulated to deplete or overexpress specific moleules known to be produced by the cells *in situ*.
